# Influence of medical care tasks on subjective burden and gain among older adults’ family caregivers: structural equation modeling for testing the role of formal and informal support

**DOI:** 10.1186/s12877-023-04348-5

**Published:** 2023-10-06

**Authors:** Yoko Sugihara, Hidehiro Sugisawa

**Affiliations:** 1 Department of Urban Science and Policy, Faculty of Urban Environmental Sciences, Tokyo Metropolitan University, 1-1 Minami-Osawa, Hachioji-shi, Tokyo, 192-0397 Japan; 2 Gerontology Program, J. F. Oberlin University Graduate School of International Studies, 1-1- 12 Sendagaya, Shibuya-ku, Tokyo, 151-0051 Japan

**Keywords:** Medical care tasks, Family caregivers, Burden, Gain, Home medical care service

## Abstract

**Background:**

The number of caregivers performing medical care tasks at home for older adults is expected to increase. Family caregivers, who are not healthcare professionals, are likely to find these activities difficult and burdensome. However, appropriate support may decrease the negative and increase the positive aspects of caregiving. This study investigated direct associations between caregivers providing medical care at home and their negative and positive appraisals of caregiving (burden and gain), indirect associations through healthcare professional support and informal support, and whether the associations between medical care tasks and caregivers’ appraisals of caregiving differed based on the support received.

**Methods:**

Interview surveys were conducted in 2013, 2016, and 2019 in a Tokyo Metropolitan Area city with family caregivers of community-dwelling older adults who were certified as requiring care in Japan’s long-term care insurance system. This study analyzed the combined data from each survey (n = 983). Structural equation modeling (SEM) analysis was utilized to examine direct associations between providing medical care and caregiver appraisals and indirect relationships through healthcare professional support and informal support. The modulating effects of these forms of support on the relationship between medical care and caregiver appraisals were assessed using multigroup SEM analyses.

**Results:**

Approximately 9% of family caregivers provided medical care at home. The results of SEM analyses, controlled for care recipients’ physical and cognitive difficulties; caregivers’ age, sex, and economic condition; and survey year, revealed no direct associations between providing medical care and caregivers’ sense of burden and gain. They also did not reveal any indirect effects through either healthcare professional support or informal support. However, the results of multigroup SEM analyses indicated that caregivers providing medical care who used home-visit services by physicians and/or nurses, compared to those who did not, tended to exhibit a greater sense of gain.

**Conclusions:**

These results suggest that family caregivers providing medical care at home can positively change their appraisals of caregiving if they receive appropriate support. Home medical care services provided by healthcare professionals can effectively support caregivers. Developing strategies and policies to make medical care services at home more accessible to caregivers is crucial.

## Background

Recent global trends toward earlier hospital discharges have shifted care into the hands of the community for individuals with medical care needs, especially older adults [1–3]. Consequently, family caregivers are increasingly performing medical care tasks at home for adult care recipients, in addition to personal care and household chores [4–6]. Medical care tasks are complex and skilled activities requiring medical knowledge and judgment [5]. These tasks, including suctioning, respiratory management, and wound care, must be performed both during the day and at night [5]. However, healthcare service providers are only present intermittently at home; therefore, family caregivers are frequently required to perform medical care tasks [7]. Moreover, professional healthcare services are expensive [8], and hence, family caregivers may be compelled to perform these tasks for financial reasons [9].

Family caregivers, who are not healthcare professionals, are likely to find performing these tasks throughout the day difficult and burdensome. Nevertheless, the current knowledge about what types of medical care tasks are performed at home, the number of caregivers performing these tasks, and the impact of these activities on caregivers’ physical and mental health is limited [10, 11]. However, recent studies in the U.S. have demonstrated that family caregivers who perform medical care tasks exhibit higher risks of emotional and physical strain, burden, sleep disturbances, financial difficulty, restricted participation in valued activities, and loss of work productivity than those who do not [1, 6, 10–13].

By contrast, in a study examining spouses of older adults with functional disabilities, a higher number of medical/nursing tasks is significantly linked to greater caregiving gains [11]. Caregiving gains include any positive affective or practical return derived from providing care, such as a sense of fulfillment, rewards, feeling useful, learning new skills, confidence in coping with challenges, and feeling closer to the care recipient [14–17]. Polenick et al. [11] indicated that performing medical/nursing tasks may increase caregivers’ exposure to opportunities for personal growth, such as successfully coping with challenges and learning new skills, thereby enhancing the positive aspects of caregiving. Numerous studies, especially those on dementia caregiving experience, have demonstrated that family caregivers perceive negative as well as positive aspects of caregiving [14, 17–25], and similar results have been reported in Japan [26–29]. Hence, even in challenging situations, such as performing medical care tasks at home, family caregivers may perceive both the negative and positive aspects of caregiving. However, few empirical studies have examined the positive effects of these activities.

Understanding the factors that reduce negative consequences and promote positive experiences in difficult situations is imperative to improving caregivers’ quality of life. Numerous studies have attempted to identify factors associated with negative or positive outcomes for caregivers based on Pearlin et al.’s stress process model [30]. These factors are categorized into (a) background/contextual factors, including age, sex, ethnicity, kinship, income, educational level, and cohabitation; (b) primary stressors, including care recipients’ dependencies pertaining to activities of daily living (ADL) or instrumental ADL (IADL), cognitive impairments, and problem behaviors; and (c) mediators, including coping strategies and social support [18, 23–25, 31–40]. However, existing knowledge about the factors associated with less negative and more positive aspects of caregiving among family caregivers performing medical care tasks at home is limited.

Polenick et al. [11] reported that medical care activities may precipitate both positive and negative consequences for spousal caregivers, depending partially on sociodemographic characteristics. Regarding the sociodemographic characteristics of caregivers providing medical care at home, previous studies have indicated that caregivers who are wives, less educated [11], and older [10] are linked with negative consequences. Additionally, primary stressors, such as caregivers’ health problems [10] and care recipients’ cognitive impairments [12, 13, 41], are reportedly associated with negative outcomes for caregivers providing medical care at home. Although sociodemographic characteristics and primary stressors are significant domains in the caregiver stress process model, we must consider the role of mediators that may reinforce or attenuate the strength of the relationship between stressors and outcomes. Social support is useful in considering effective interventions for caregivers and, hence, is a crucial mediator [30, 34, 35]. However, knowledge about the role of formal and informal support in the context of caregivers providing medical care at home is limited.

Regarding formal support, some studies have demonstrated that family caregivers often do not receive training for performing medical care tasks [3, 5, 42], and several of these caregivers do not use support services [6, 42]. Meanwhile, recent studies have suggested that adequate support and training from healthcare professionals are associated with a lower likelihood of acute care utilization and reduced caregiver burden [43, 44]. Therefore, support from healthcare professionals may be effective in alleviating the negative outcomes of caregivers providing medical care at home. However, information about the relationship between support from healthcare professionals and positive outcomes for caregivers who provide medical care at home is scant.

Additionally, informal support from other family members or friends is considered a crucial resource for caregivers; however, minimal consensus exists regarding the relationship between informal support and caregivers’ appraisals of caregiving. A recent systematic review with meta-analysis on the effects of social support on subjective burden among informal caregivers of adults and older adults concluded that perceived support may be a good predictor of subjective burden, while actual received support is not [45]. The relationship between received support and caregiver burden is complex, and the effectiveness of support for caregivers may vary depending on the caregiving situation. Regarding caregivers providing medical care at home, the actual support received may be effective because it is burdensome for only one caregiver to perform these tasks.

Considering that the number of family caregivers performing medical care tasks at home is expected to increase in the future [4–6], understanding the influence of medical care activities on caregivers and identifying formal or informal support that reduce negative impacts and increases the positive impacts would be useful in developing policies for in-home care. Japan is a super-aged society where approximately 30% of the population is over 65 years old, the highest percentage in the world [46], and care for older adults is a critical social issue. Since family members often live with and care for older adults in Japan [47], studies in the country may provide meaningful insights into the influences of family members providing medical care at home.

This study aims to investigate (i) the direct associations between caregivers performing medical care tasks at home and their negative (i.e., burden) and positive (i.e., gain) appraisals of caregiving; (ii) the indirect associations through support from healthcare professionals and informal support; and (iii) whether the associations between medical care tasks and caregiver appraisals vary by support from healthcare professionals and informal support. This study examines the role of support from the following two perspectives: the mediating effect (second research objective) and the modulating effect (third research objective). Pearlin et al. [30] suggested that the mediators in the stress process model may serve both to block their contagion at the junctures between the primary stressors and secondary role strains (i.e., mediating effect) and to attenuate or reinforce the strength of the relationships between stressors and outcomes (i.e., modulating effect). Therefore, this study examines both the mediating and the modulating effects of support to understand the multifaceted role of support in the context of caregivers performing medical care tasks at home.

## Methods

### Study design and participants

The public long-term care insurance system was introduced in 2000 in Japan. Under this system, people requiring long-term care or support are classified into seven levels based on their care needs and are registered on a list maintained by each local government. People with high medical or nursing care needs are certified as Care levels 1–5; the higher the number of care levels, the higher the need for long-term care.

First, we identified 1,000 individuals aged 65 years or older requiring medical or nursing care by randomly selecting 200 certified individuals from each category of Care levels 1–5, excluding nursing home residents, in August 2013, in a Tokyo Metropolitan Area city. Second, face-to-face interviews were conducted with family caregivers of older adults requiring medical or nursing care in their homes between September and October 2013. Using the same procedure, repeated cross-sectional surveys were administered in 2016 and 2019. The survey response rates were 67.5, 65.7, and 62.0% in 2013, 2016, and 2019, respectively.

The surveyed city is located in the center of Tokyo. According to the 2015 census [48], the aging rate in this city was 21.2%, lower than the national rate in Japan (26.6%) but similar to that in Tokyo as a whole (22.7%). Furthermore, the percentage of single-person households among households with a person aged 65 and over was 34.4%, higher than the national percentage (27.3%) but similar to that of Tokyo as a whole (35.8%). The average percentage of those certified as needing care or support in the older population during the survey period (2013–2019) was 18.7%, similar to the national percentage and that of Tokyo as a whole (18.0% and 18.3%, respectively) [49].

For this study’s analyses, participants were selected as family caregivers who primarily cared for older adults at home. Considering that the number of caregivers providing medical care at home was minimal, the data for each survey were combined. The participants of each survey were independent, with no overlaps. The distribution of the variables used in the analyses did not differ significantly by the survey year. After excluding those with missing values for the variables used in the analyses (5.2% of participants), the final sample comprised 983 participants (n = 356, n = 331, and n = 296 from the 2013, 2016, and 2019 surveys, respectively).

### Measurements

#### Caregiver burden

Caregiver burden was measured using the Caregivers’ Subjective Burden Scale [50]. This scale comprises eight items measuring the intensity of perceived caregiver burden on the difficulties caused by caregiving, including physical and mental health problems, conflicts between work and caregiving, conflicts between housework or childcare and caregiving, restriction on going out, restriction on free time, family conflicts, and financial issues. Response categories range from 0 (not at all) to 3 (very much), with higher scores indicating a greater subjective burden. The scale’s internal consistency (Cronbach’s alpha) was 0.855. Caregiver burden was operationalized as a latent construct inferred from the aforementioned eight items.

#### Caregiver gain

Caregiver gain was measured using the Caregivers’ Personal Attainment Scale [50]. This scale comprises five items measuring subjective feelings of satisfaction and rewards resulting from caregiving, including feelings of fulfillment, confidence, appreciation from the care recipient, emotional connection with the care recipient, and happiness. The response categories range from 0 (strongly disagree) to 4 (strongly agree), with higher scores indicating greater subjective gain. The scale’s internal consistency (Cronbach’s alpha) was 0.760. Caregiver gain was operationalized as a latent construct inferred from the aforementioned five items.

### Medical care tasks

Medical care tasks were identified based on whether the participant provided any of the following types of care to the care recipient (1 = yes, 0 = no): pressure ulcer care, nasogastric tube feeding, gastrostomy/enterostomy tube feeding, suctioning, oxygen therapy, tracheotomy, injections, dialysis care, self-catheterization, indwelling bladder catheter, and other medical treatments.

### Support from healthcare professionals and informal support

Support from healthcare professionals was identified by whether the participant used home medical care services, such as a doctor’s visit at home or home-visit nursing service (1 = yes, 0 = no). Informal support was measured based on the response to the question, “If you (the respondent) are unable to care for the care recipient due to urgent matters or other reasons, do you have a family member, relative, or friend who can take over your caregiving responsibilities for approximately one week?” (1 = yes, 0 = no). This question not only refers to the experience of having someone take over caregiving responsibilities for approximately one week but also includes the possibility of such a situation.

#### Covariates

The covariates included in the analyses were the care recipient’s ADL dependencies and memory-related and behavioral problems, and the caregiver’s age (in years), sex (0 = male caregiver, 1 = female caregiver), subjective economic condition (1 = quite difficult to 5 = comfortable), and survey year.

The care recipients’ degree of ADL dependency was measured by whether they depended on help from others for five basic ADLs (walking, eating, dressing, bathing, and toileting). Each item was rated from 0 (independent) to 3 (need for full assistance), with higher scores indicating higher dependency. This scale’s internal consistency (Cronbach’s alpha) was 0.882. The degree of ADL dependency was operationalized as a latent construct inferred from these five items.

The degree of memory-related and behavioral problems was measured using the Checklist for Dementia Rating in Japanese community residents [51] to assess the care recipient’s memory losses, recognition failures, and socially inappropriate behaviors. The response categories are dichotomized (0 = absence, 1 = presence); this 16-item scale ranges from 0 to 16, with higher scores indicating greater memory-related and behavioral problems. Examples of items are “difficulty remembering his/her age,” “difficulty recognizing relatives living with him/her,” “agitation at night,” and “putting non-food items in the mouth.” The internal consistency (Cronbach’s alpha) was 0.907.

### Statistical approach

First, structural equation modeling (SEM) was used to examine whether performing medical care tasks was directly related to caregiver burden and gain and whether these relationships were mediated by support from healthcare professionals and informal support. The criteria for the goodness of the model-data fit were comparative fit index (CFI) and Tucker–Lewis Index (TLI) ≥ 0.95, standardized root-mean-square residual (SRMR) ≤ 0.08, and root-mean-square error of approximation (RMSEA) ≤ 0.06 [52]. Bootstrapping was used to test the mediating effect (5,000 re-samples), wherein the effect was deemed significant with a 95% bias-corrected confidence interval (CI), not including zero [53].

Second, the modulating effects of support from healthcare professionals and informal support were assessed for differences in the path coefficients from performing medical care tasks to caregiver appraisals (burden and gain) across the two groups (caregivers with support vs. without support); to this end, multigroup SEM analyses were performed, wherein the invariant model was compared with an unconstrained model [54]. The invariant model constrained factor loadings, structural path coefficients, covariances, and residuals to be equal across the two groups. The unconstrained model allowed for the free estimation of all parameters across the two groups. The chi-square difference test (Δχ^2^), CFI, TLI, SRMR, RMSEA, and Akaike information criterion (AIC) were used to compare model fit between the two models. If a better model fit was obtained from the unconstrained model, the critical ratios for differences were estimated to compare path coefficients across groups. The data were analyzed using SPSS 25 and AMOS 25.

## Results

### Descriptive statistics and correlation analysis

Table 1 presents descriptive statistics and intercorrelations for the measures under study. The primary family caregivers’ average age was 67.3 years, and 71.1% were female. Of the participants, 9.2% of caregivers performed medical care tasks. The most frequently performed medical care task was pressure ulcer care (44.4%), followed by suctioning (23.3%) and gastrostomy/enterostomy tube feeding (17.8%). Many of the medical care tasks had to be performed regularly. Furthermore, 36.3% of caregivers used home medical care services, such as a doctor’s visit or home-visit nursing service, and 43.9% had an informal supporter who provided care on their behalf for approximately one week. Regarding demographic characteristics, caregivers who performed medical care tasks at home, compared to those who did not, were more likely to be wives (38.9% vs. 28.2%); furthermore, a higher proportion of them were women (75.6% vs. 70.7%), but their average ages were similar (67.0 years vs. 67.3 years).

**Table 1:** Descriptive statistics and intercorrelations for the variables under study (n = 983)

	Mean	(SD)	Intercorrelations									
	or %		1	2	3	4	5	6	7	8	9	10
1. Caregiver burden	9.63	(5.71)	1									
2. Caregiver gain	11.13	(3.94)	− 0.19***	1								
3. Medical care tasks	9.2%		0.06	0.09**	1							
4. Home medical care services	36.3%		0.04	0.11***	0.17***	1						
5. Informal support	43.9%		− 0.24***	0.08**	− 0.05	− 0.02	1					
6. CR’s ADL dependencies	7.56	(4.41)	0.25***	0.14***	0.24***	0.40***	− 0.11	1				
7. CR’s MB problems	2.23	(3.15)	0.25***	− 0.10***	− 0.01	0.01	− 0.04	0.18***	1			
8. Caregiver’s age	67.28	(11.88)	− 0.13***	0.05	− 0.01	0.01	− 0.05	− 0.02	− 0.05	1		
9. Female caregiver	71.1%		0.19***	− 0.05	0.03	− 0.09**	− 0.11***	− 0.01	0.04	− 0.17***	1	
10. Economic condition	2.85	(0.81)	− 0.30***	0.16***	0.06*	− 0.02	0.13***	− 0.09**	− 0.09**	0.01	− 0.05	1

* *p* < 0.05, ** *p* < 0.01, *** *p* < 0.001

CR, care recipient; ADL, activities of daily living; MB, memory-related and behavioral; SD, standard deviation

Bivariate correlation analyses and t-tests for the correlation coefficient revealed that performing medical care tasks was not significantly correlated with caregiver burden but was positively correlated with caregiver gain (*r* = 0.09, *p* < 0.01). Performing medical care tasks was positively associated with using home medical care services (*r* = 0.17, *p* < 0.001) but was not significantly associated with informal support. Home medical care services were not significantly related to caregiver burden but were positively related to caregiver gain (*r* = 0.11, *p* < 0.001). Informal support exhibited a significant negative relationship with caregiver burden (*r* = − 0.24, *p* < 0.001) and a positive relationship with caregiver gain (*r* = 0.08, *p* < 0.01).

### Direct, indirect, and total effects of performing medical care tasks

Table 2 presents the results of the direct, indirect, and total effects of performing medical care tasks on caregiver burden and gain, controlling for care recipients’ ADL dependencies and memory-related and behavioral problems; caregivers’ age, sex, and subjective economic condition; and survey year. The model’s goodness-of-fit indices were CFI = 0.96, TLI = 0.95, SRMR = 0.05, and RMSEA = 0.04, indicating a good fit. However, performing medical care tasks did not exhibit statistically significant direct, indirect, or total effects on caregivers’ sense of burden and gain. Performing medical care tasks exhibited a significant direct effect on the use of home medical care services (*β* = 0.09, 95%CI: 0.03, 0.15), and home medical care services were associated with lower caregiver burden (*β* = -0.05, 95%CI: -0.12, 0.02) and higher gain (*β* = 0.03, 95%CI: -0.05, 0.11); however, these relationships were not statistically significant. Regarding informal support, having a relative or others take over caregiving responsibilities for approximately one week was significantly associated with lower caregiver burden (*β* = -0.20, 95%CI: -0.26, -0.13) and higher gain (*β* = 0.07, 95%CI: 0.01, 0.14), whereas performing medical care tasks did not increase informal support (*β* = -0.04, 95%CI: -0.10, 0.02).

**Table 2:** Direct, indirect, and total effect estimates with bias-corrected bootstrap 95% confidence intervals

Structural paths	*β*	95%CI
**Direct effect**		
Medical care tasks → Burden	0.04	(-0.03, 0.10)
Medical care tasks → Gain	0.05	(-0.02, 0.12)
Medical care tasks → Home medical care services	**0.09**	**(0.03, 0.15)**
Medical care tasks → Informal support	-0.04	(-0.10, 0.02)
Home medical care services → Burden	-0.05	(-0.12, 0.02)
Home medical care services → Gain	0.03	(-0.05, 0.11)
Informal support → Burden	**-0.20**	**(-0.26, -0.13)**
Informal support → Gain	**0.07**	**(0.01, 0.14)**
**Indirect effect**		
Medical care tasks → Home medical care services → Burden	-0.01	(-0.04, 0.01)
Medical care tasks → Home medical care services → Gain	0.01	(-0.01, 0.03)
Medical care tasks → Informal support → Burden	0.02	(-0.01, 0.06)
Medical care tasks → Informal support → Gain	-0.01	(-0.03, 0.01)
**Total effect**		
Medical care tasks → Burden	0.05	(-0.04, 0.12)
Medical care tasks → Gain	0.05	(-0.03, 0.13)

The bold estimates and confidence intervals (CIs) are significant (0 is not included in the 95% bootstrap CI)

*β,* standardized path coefficient estimates; 95%CI, bias-corrected 95% bootstrap CI

The model was adjusted for care recipients’ dependencies related to activities of daily living and memory-related and behavioral problems; caregiver’s age, sex, and subjective economic condition; and survey year

Goodness-of-fit indices of the model: CFI = 0.96, TLI = 0.95, SRMR = 0.05, RMSEA = 0.04

Calculations are based on 5,000 bootstrap samples

### Modulating effects of support on the relationships between medical care and caregiver appraisals

Multigroup SEM analyses were conducted to verify whether the path coefficients in the model were similar or different across caregiver groups with/without support from healthcare professionals and with/without informal support. As presented in Table 3, the chi-square difference tests revealed significant differences between the unconstrained models (i.e., the parameters were freely estimated) and the invariant models (i.e., the parameters were constrained equally across groups) in the analyses of both caregivers using/not using home medical care services (Δ*χ*^2^(57) = 331.84, *p* < 0.001) and caregivers with/without informal support (Δ*χ*^2^(57) = 117.90, *p* < 0.001). In both analyses, the unconstrained models showed adequate model-data fit and better fit indices compared with the invariant models, indicating that some paths among the variables were modulated by support from healthcare professionals and informal support.

**Table 3:** Multigroup model comparisons

	Chi-square difference test			Goodness-of-fit indices				
	Δ*χ*^2^	Δ*df*	*p*	CFI	TLI	SRMR	RMSEA	AIC
**Caregivers using/not using home medical care services**								
Unconstrained model				0.96	0.95	0.06	0.03	1048.47
Invariant model	331.84	57	< 0.001	0.93	0.92	0.06	0.03	1266.30
**Caregivers with/without informal support**								
Unconstrained model				0.96	0.95	0.05	0.03	1091.38
Invariant model	117.90	57	< 0.001	0.95	0.95	0.06	0.03	1095.28

The unconstrained model allows free estimation of all parameters across the two groups

The invariant model constrains factor loadings, structural path coefficients, covariances, and residuals to be equal across the two groups

CFI, comparative fit index; TLI, Tucker–Lewis Index; SRMR, standardized root-mean-square residual; RMSEA, root-mean-square error of approximation; AIC, Akaike information criterion

**Fig. 1:**
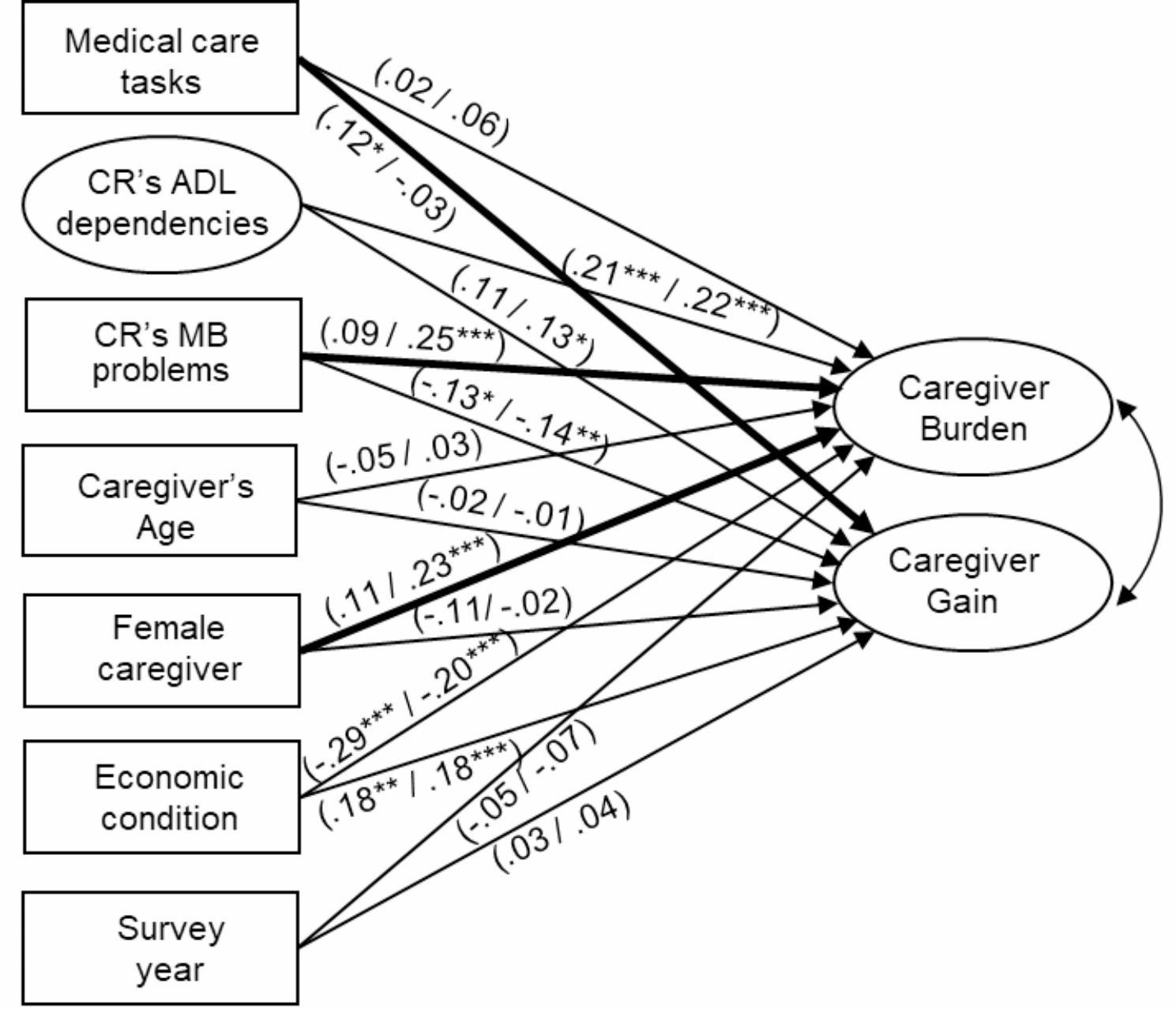
**Multigroup structural model: group comparison according to the use of home medical care services** Standardized path coefficients are indicated for caregivers using home medical care services (left in parentheses) and those not using home medical care services (right in parentheses) for the unconstrained model Bold paths represent the significant difference between the two groups * *p* < 0.05, ** *p* < 0.01, *** *p* < 0.001 For parsimony, intercorrelations among the correlates are not presented The ellipses represent latent variables. The rectangles depict observed variables Model fit indices: CFI = 0.96, TLI = 0.95, SRMR = 0.06, RMSEA = 0.03 CR, care recipient; ADL, activities of daily living; MB, memory-related and behavioral

Figure 1 presents the results of the group comparisons pertaining to the use of home medical care services. The critical ratio for differences revealed significant divergences between caregivers who used home medical care services and those who did not in the following three structural path coefficients: the path from performing medical care tasks to caregiver gain, the path from the care recipient’s memory-related/behavioral problems to caregiver burden, and the path from the caregiver’s sex to caregiver burden. Among these modulating effects of home medical care services, regarding medical care tasks, the path coefficient from performing medical care tasks to caregiver gain tended to exhibit a significant z-test statistic between the two groups (*Z* = -1.78, *p* < 0.10). Regarding the standardized path coefficients and *p*-values for the significance of the t-test, performing medical care tasks was positively related to caregiver gain among caregivers who used home medical care services (*β* = 0.12, *p* < 0.05) but not among those who did not use such services (*β* = -0.03, *p* = 0.55).

**Fig. 2:**
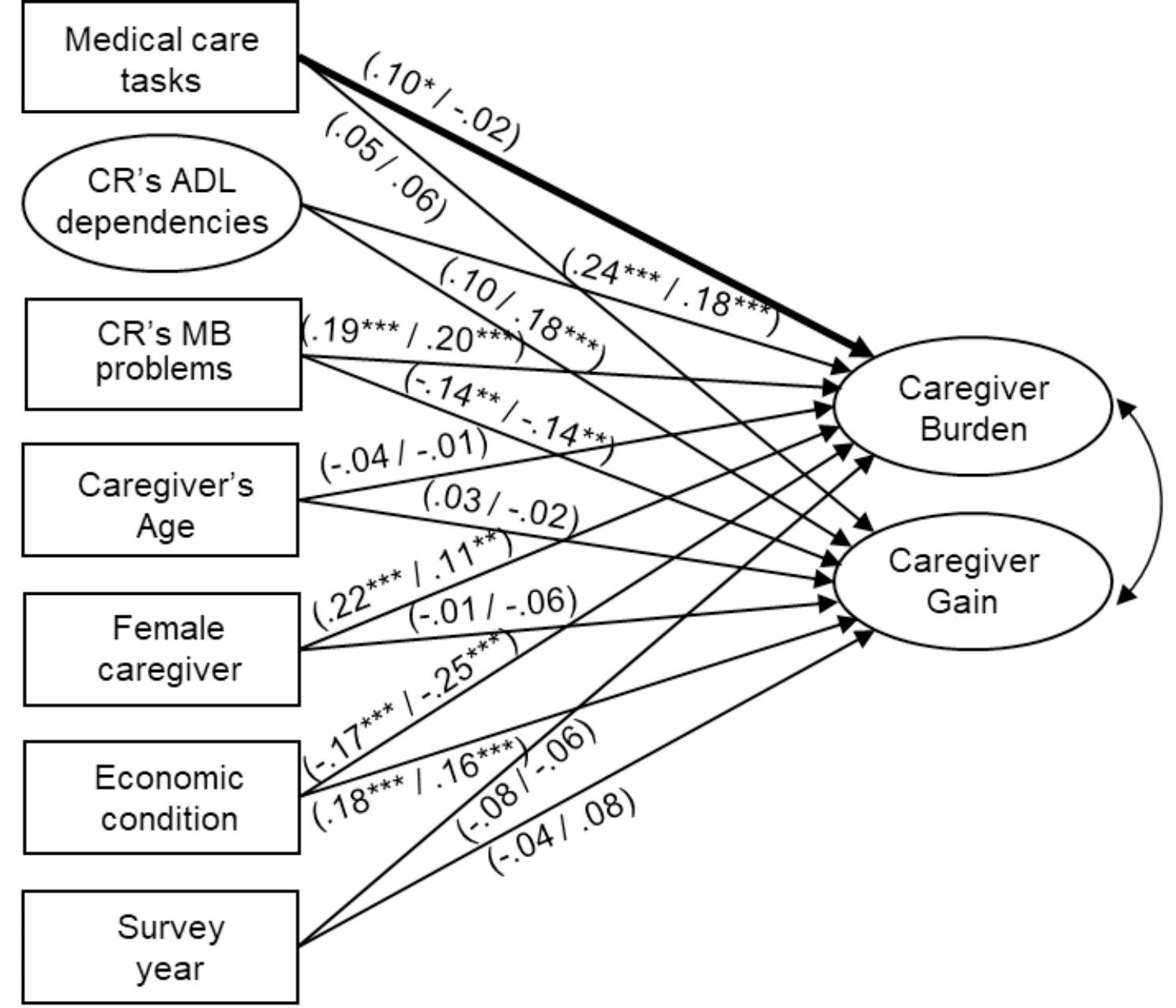
**Multigroup structural model: group comparison according to the use of informal support** Standardized path coefficients are indicated for caregivers with informal support (left in parentheses) and caregivers without informal support (right in parentheses) for the unconstrained model Bold paths represent the significant difference between the two groups * *p* < 0.05, ** *p* < 0.01, *** *p* < 0.001 For parsimony, intercorrelations among the correlates are not presented The ellipses represent latent variables. The rectangles depict observed variables Model fit indices: CFI = 0.96, TLI = 0.95, SRMR = 0.05, RMSEA = 0.03 CR, care recipient; ADL, activities of daily living; MB, memory-related and behavioral

Figure 2 depicts the results of the group comparisons pertaining to having or not having informal support. The critical ratio for differences revealed that only the structural path coefficient from performing medical care tasks to caregiver burden tended to exhibit a significant z-test statistic between the two groups (*Z* = -1.97, *p* < 0.05). Regarding the standardized path coefficient and *p*-value for the significance of the t-test, performing medical care tasks was positively related to caregiver burden among caregivers with informal support (*β* = 0.10, *p* < 0.05) but not among caregivers without informal support (*β* = -0.02, *p* = 0.69).

## Discussion

This study revealed that family caregivers providing medical care at home positively change their appraisals of caregiving if they receive appropriate support. Caregivers performing medical care tasks at home who used home-visit services provided by physicians and/or nurses, compared to those who did not, tended to exhibit a greater sense of gain. This study’s results demonstrated that home medical care services provided by healthcare professionals are an effective means of support for family caregivers of older adults with medical care needs.

Although we hypothesized that performing medical care tasks at home would exhibit both negative and positive effects on family caregivers, no direct associations between performing medical care tasks and caregivers’ sense of burden and gain were found. Considering that numerous people needing medical care have severe physical or cognitive disabilities, the impact of medical care tasks on caregiver appraisals may overlap with the impact of such disabilities. Thus, in the multivariate analyses that controlled for care recipients’ ADL dependencies and memory-related/behavioral problems, medical care tasks might not have demonstrated a statistically significant association with caregiver appraisals. This hypothesis may be particularly consistent with caregiver gain because medical care tasks were positively correlated with caregiver gain in the bivariate analysis, but there was no association in the multivariate analysis controlling for ADL dependencies and memory-related/behavioral problems.

By contrast, caregiver burden did not exhibit a statistically significant association with medical care tasks even in the bivariate analysis, suggesting that medical care tasks may be associated with more positive appraisals of caregiving than negative appraisals. Caregivers performing medical care tasks at home may be highly motivated to provide care to family members and have a better environment for providing care at home. In Reinhard et al.’s [42] study, caregivers who performed multiple medical care tasks were more likely to report that family caregiving activities prevented family members from moving to a nursing home. Because acquiring advanced caregiving skills, such as medical care, can increase caregivers’ confidence and efficacy, performing medical care tasks may positively impact caregivers.

Regarding mediating effects, no statistically significant indirect effects were found for both support from healthcare professionals and informal support. However, in terms of support from healthcare professionals, caregivers who provided medical care were more likely to use home medical care services; this support slightly reduced caregivers’ burden and increased their gain. Regarding modulating effects, a promoting effect of home medical care services on caregiver gain was confirmed; caregivers performing medical care tasks at home who used home medical care services, compared to those who did not, tended to exhibit a greater sense of gain. These results suggest that support from healthcare professionals may change caregivers’ appraisals of caregiving from negative to positive. Previous studies on cancer patients’ caregivers have suggested that medical care skill training improves caregivers’ confidence and self-efficacy [55, 56]. Similarly, for caregivers of older adults with medical care needs, receiving advice and training on medical care tasks from physicians and nurses may increase caregiver gain, including confidence and a sense of accomplishment.

Although support from healthcare professionals, including explanations of symptoms and coping methods, is effective for family caregivers, several studies have indicated that family caregivers often do not receive training for medical care tasks [3, 5, 42], and several of them do not use support services [6, 42]. Home medical care services provided by healthcare professionals are difficult to use frequently because of their high fees. However, according to this study’s findings, home medical care services are effective for caregivers performing medical care tasks and dementia care at home. Therefore, developing strategies and policies to make medical care services at home more accessible to caregivers is crucial.

By contrast, informal support did not increase even when caregivers performed medical care tasks at home, and caregivers with informal support felt more burdened than those without it. Considering that medical care tasks require specialized skills and knowledge, providing help is difficult for laypersons, and informal support may not increase even if caregivers perform medical care tasks at home. Even if informal supporters are available, they may exacerbate the caregiver’s burden if they are unfamiliar with medical care tasks. Although this study did not find mediating or buffering effects of informal support on the relationship between medical care tasks and caregiver appraisals, informal support was shown to be directly related to lower caregiver burden and higher gain, thereby suggesting that informal support is also important in situations where providing medical care is not considered.

This study had several limitations. First, because of the cross-sectional nature of the study, causal associations could not be determined. Second, since only 9.2% (n = 90) of caregivers in this study performed medical care tasks at home, examining, for instance, the differences in effects by type and frequency of support was difficult when analyzing the mediating and buffering effects of support on the relationships between providing medical care and caregivers’ sense of burden and gain. Furthermore, considering the combined effects of formal and informal support on caregivers is important; however, this could not be clarified in this study given the small number of caregivers providing medical care at home. Third, the surveys were conducted only in urban areas of Japan; a survey on caregivers in rural areas may yield different findings.

## Conclusions

This study confirmed that professional healthcare support, such as home-visit services by physicians and/or nurses, can potentially increase positive appraisals of caregiving among caregivers performing medical care tasks at home. This study’s results indicate that appropriate support from healthcare professionals can improve caregivers’ appraisals of caregiving. The findings can help enhance policies pertaining to caregivers performing medical care tasks at home—a trend that is expected to rise in the future.

## Data Availability

The datasets analyzed in this study are available from the corresponding author upon reasonable request.

## Abbreviations

ADL: Activities of Daily Living
IADL: Instrumental Activities of Daily Living
SEM: Structural Equation Modeling
CFI: Comparative Fit Index
TLI: Tucker–Lewis Index
SRMR: Standardized Root-Mean-Square Residual
RMSEA: Root-Mean-Square Error of Approximation
CI: Confidence Interval
AIC: Akaike Information Criterion
